# Down-regulation of DNA key protein-FEN1 inhibits OSCC growth by affecting immunosuppressive phenotypes via IFN-γ/JAK/STAT-1

**DOI:** 10.1038/s41368-023-00221-8

**Published:** 2023-04-25

**Authors:** Shimeng Wang, Xiangjian Wang, Jun Sun, Jin Yang, Deyang Wu, Fanglong Wu, Hongmei Zhou

**Affiliations:** 1 State Key Laboratory of Oral Diseases & National Center of Stomatology & National Clinical Research Center for Oral Diseases & Frontier Innovation Center for Dental Medicine Plus & Department of Oral Medicine, West China Hospital of Stomatology, Sichuan University, Chengdu, China; 2 Department of Oral Medicine, The Second Affiliated Hospital of Zhejiang University School of Medicine, Zhejiang University, Hangzhou, China; 3 Department of Oral Medicine, Stomatological Hospital of Chongqing Medical University, Chongqing Medical University, Chongqing, China

**Keywords:** Oral cancer, Tumour biomarkers

## Abstract

Oral squamous cell carcinoma (OSCC) escape from the immune system is mediated through several immunosuppressive phenotypes that are critical to the initiation and progression of tumors. As a hallmark of cancer, DNA damage repair is closely related to changes in the immunophenotypes of tumor cells. Although flap endonuclease-1 (FEN1), a pivotal DNA-related enzyme is involved in DNA base excision repair to maintain the stability of the cell genome, the correlation between FEN1 and tumor immunity has been unexplored. In the current study, by analyzing the clinicopathological characteristics of FEN1, we demonstrated that FEN1 overexpressed and that an inhibitory immune microenvironment was established in OSCC. In addition, we found that downregulating FEN1 inhibited the growth of OSCC tumors. In vitro studies provided evidence that FEN1 knockdown inhibited the biological behaviors of OSCC and caused DNA damage. Performing multiplex immunohistochemistry (mIHC), we directly observed that the acquisition of critical immunosuppressive phenotypes was correlated with the expression of FEN1. More importantly, FEN1 directly or indirectly regulated two typical immunosuppressive phenotype-related proteins human leukocyte antigen (HLA-DR) and programmed death receptor ligand 1 (PD-L1), through the interferon-gamma (IFN-γ)/janus kinase (JAK)/signal transducer and activator transcription 1 (STAT1) pathway. Our study highlights a new perspective on FEN1 action for the first time, providing theoretical evidence that it may be a potential immunotherapy target for OSCC.

## Introduction

According to the latest report published in 2020, of 377 713 new cases of oral cancer, 177 757 led to death, and among these mortality cases, 90% were attributed to OSCC.^1,2^ Notably, the 5-year survival rate for OSCC is approximately 50%, and high mortality and recurrence rates are problems that urgently need to be solved.^3,4^ Immune escape, a phase in the “tumor immunoediting” hypothesis, refers to tumor cells escaping recognition and attack by the body’s immune system.^5^ In this phase, tumor cells can mediate immune escape mediated by a series of acquired immunophenotypes. HLA/major histocompatibility complex (MHC) and PD-L1 are two representative immunophenotype proteins that mediate tumor cell immune escape.^6,7^ Studies have suggested that downregulation of HLA/MHC expression reduces the immunogenicity of tumor cells, which leads to T-cell activation,^8^ while upregulating PD-L1 expression leads to T-cell immunosuppression.^9^ These hypotheses suggest that intervention of the acquisition of two important aforementioned phenotypes may play a critical role in tumor immunoediting. Therefore, means to select effective intervention pathways has become a key issue in cancer immunotherapy.

In our previous study, by combining systematic biology and cell molecular biology techniques, we successfully predicted that FEN1 might be a key cancer suppressor targets in the head and neck squamous cell carcinoma (HNSCC) protein network.^10^ FEN1 is at the core of cellular DNA metabolism,^11^ therefore, studies have mainly focused on DNA metabolism mechanisms, interactions with other proteins and gene mutations.^12–14^ FEN1 has been shown to be expressed at low levels in quiescent cells but increased in proliferating tissues.^15^ This proliferative activity-related property has also been noted in cancer cells, where FEN1 overexpression has been associated with accelerated progression and shorter survival periods.^16–19^ Notably, an abnormal DNA damage repair process not only induces the production of neoantigens on the cell surface,^8^ but also affects PD-L1 expression through the JAK/STAT1 signaling pathway and interferon stimulator genes (ISGs),^20,21^ raising the question of whether interfering with FEN1 gene expression can simultaneously affect PD-L1 and HLA/MHC levels.

Through technological advancements made in the last two decades, understanding of how the immune system can be targeted with immune checkpoints to provide clinical benefit has increased. In this context, we studied the possibility that FEN1 is an immune agent target from multiple perspectives. Starting with an exploration into the function of FEN1, we verified that FEN1 downregulation caused DNA damage in OSCC cells. More importantly, we found that FEN1 expression intervention might lead to changes in OSCC immunophenotypes. In this study, we further explored the possible mechanism of FEN1 action in the immune escape of OSCC cells and thus suggest new ideas to study FEN1 as a potential biomarker for blocking immune escape by affecting immunophenotype acquisition in the OSCC context.

## Results

### Clinicopathological characteristics of FEN1 in normal tissues, oral leukoplakia (OLK) and OSCC

**Fig. 1:**
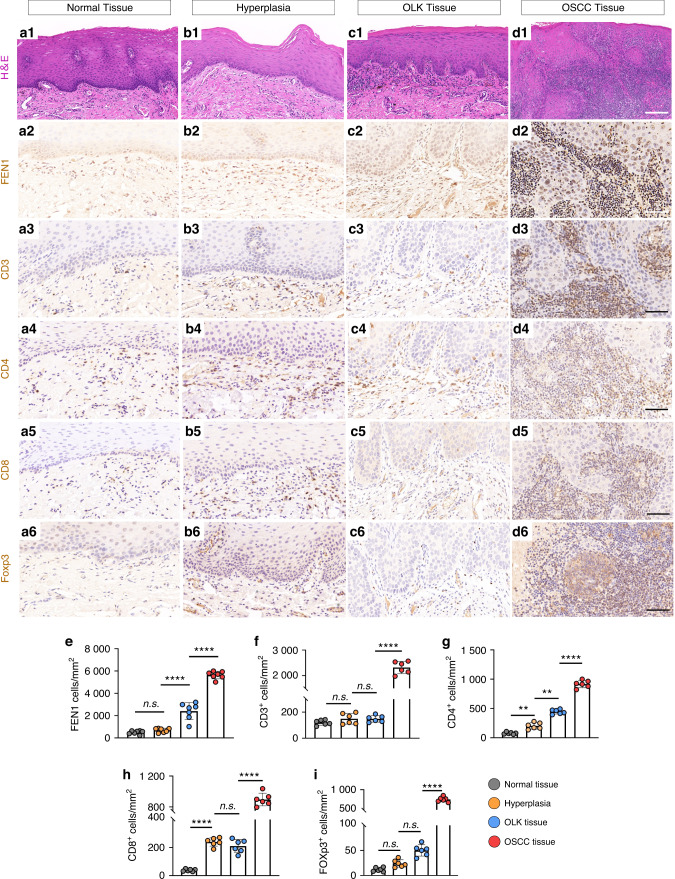
FEN1 and immune related markers expression in different tissues. **a1**–**d1** H&E staining of normal tissue, OLK and OSCC tissue. Scale bar: 200 μm. **a2**–**d2** FEN1 staining on normal tissue, OLK and OSCC tissue. **a3**–**d3** CD3^+^ staining on normal tissue, OLK and OSCC tissue. **a4**–**d4** CD4^+^ staining on normal tissue, OLK and OSCC tissue. **a5**–**d5** CD8^+^ staining on normal tissue, OLK and OSCC tissue. **a6**–**d6** Foxp3^+^ staining on normal tissue, OLK and OSCC tissue. Scale bar: 100 μm. **e**–**i** Quantification of FEN1, CD3^+^, CD4^+^, CD8^+^ and Foxp3^+^ expression level in normal, OLK and OSCC tissue. *n* = 6; error bars, mean ± SD, n.s., not significant; **P < 0.01, ****P < 0.000 1; by *t* test

To explore the expression of FEN1 in oral tissues, we collected 132 clinical specimens: 91 OSCC, 28 OLK and 13 normal oral mucosal tissue samples. Typical immunohistochemical (IHC) staining was performed for evaluating the expression of FEN1 in clinical tissues, and the results are shown in Fig. 1. IHC staining demonstrated that FEN1 was expressed at low levels in hyperplasia and OLK tissues.(Fig. 1b2, c2, Supplementary Table 1). However, the staining of FEN1 was intense in OSCC group and was observed mainly in stromal and epithelial cells (Fig. 1d2, Supplementary Table 2).

Verifying the infiltration of T lymphocytes into different tissues, our results showed that the expression of CD3^+^ T cells, CD4^+^ T cells, CD8^+^ T cells and Foxp3^+^ cells was low in hyperplasia and OLK samples (Fig. 1b3–d6, Fig. 1c3–c6). Meanwhile, the staining of CD3^+^, CD4^+^, CD8^+^ were diffusely distributed in the OSCC samples, as shown in Fig. 1d3–d5, and the infiltration was located mainly in the basal layer, forming a dense lymphocyte infiltration zone. Moreover, the Foxp3^+^ stained area was significantly increased in OSCC tissues compared to others(Fig. 1d6).

### Effects of FEN1 in proliferation, cell cycle, apoptosis and migration of OSCC

**Fig. 2:**
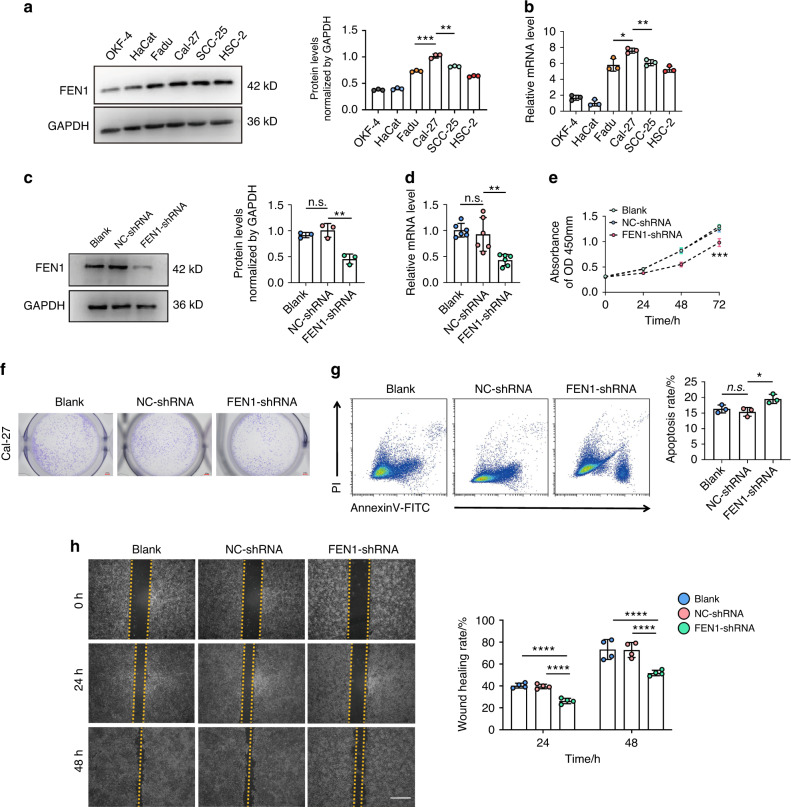
FEN1 affects multiple biological behaviors of OSCC. **a**, **b** FEN1 expression level was verified by protein and mRNA level. **c**, **d** FEN1 downregulation was confirmed through WB and qRT-PCR. **e**, **f** CCK-8 assay and colony formation were used to verify the proliferation ability of Cal-27 cells. Scale bar: 100 px. **g** Cell apoptosis assay showed the average apoptosis of Cal-27 cells increased by knocking down FEN1. **h** Representative images of wound-healing assays at the indicated times. Scale bar: 200 μm. The relative breadth of the wound after migration was measured and calculated among the groups. *n* = 4; error bars, mean ± SD, n.s., not significant; *P < 0.05, **P < 0.01, ***P < 0.001, ****P < 0.000 1; by *t* test or two-way ANOVA

The expression level of FEN1 in different OSCC lines was verified by protein level and mRNA level respectively (Fig. 2a, b). Among them, the FEN1 expression level in Cal-27 cells was the highest and significantly different from other cell groups. Therefore, Cal-27 cells were selected in the follow-up experiment. Subsequently, we knocked down FEN1 expression levels in Cal-27 cells (called FEN1-shRNA group) to further validate biological behavior in vitro (Fig. 2c, d).

Cell counting kit-8 (CCK-8) assay showed that FEN1 knockdown suppressed the proliferation of Cal-27 cells (Fig. 2e). Colony formation assay also demonstrated that the colony formation of Cal-27 cells was inhibited by FEN1 down-regulating (Fig. 2f). By flow cytometry (Fig. 2g), we found that the average apoptosis rate of Cal-27 cells increased significantly after FEN1 knockdown. In the meantime, cell cycle assay showed that the proportion of G0/G1 phase in Cal-27 cells increased by FEN1 down-regulating, while the proportion of S phase and G2/M phase decreased (Supplementary Fig. 1). Additionally, wound-healing assay provided the evidence that FEN1 knockdown attenuated the migration ability of Cal-27 cells compared with the controls (Fig. 2h).

### Down-regulation of FEN1 inhibited tumor growth through promoting DNA damage

**Fig. 3:**
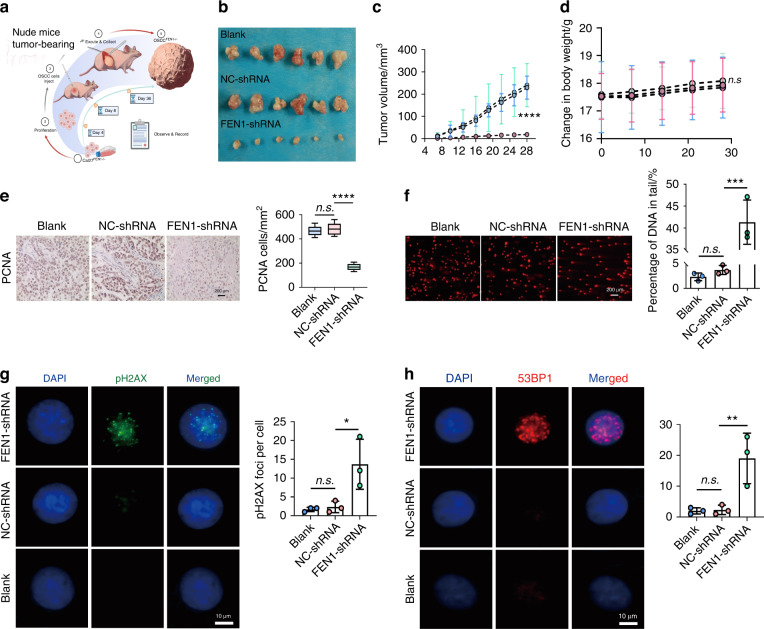
FEN1 knockdown in Cal-27 suppresses tumor growth in vivo and cause DNA damage. **a** Schematic diagram of xenograft mouse models in vivo. **b**, **c** tumors were measured with calipers, and volume was calculated every 7 days, and the line chart shows the differences (*n* = 6). **d** mice weight was measured and documented every week (*n* = 6). **e** Representative immunohistochemistry staining of PCNA in tumors of mouse models. Scale bar: 200 μm. *n* = 6; error bars, mean ± SD; by *t* test. **f** Comet assay demonstrated that FEN1 knockdown could increase the percentage of Tail DNA (×200) Scale bar: 200 μm. **g**, **h** immunofluorescence showed FEN1 downregulation could induce DNA double strain damage, the expression of pH2AX and 53BP1 significantly increased, forming foci (×400) Scale bar: 200 μm. *n* = 3; error bars, mean ± SD, n.s., not significant; *P < 0.05, **P < 0.01, ***P < 0.001, ****P < 0.000 1; by *t* test or two-way ANOVA

To verify the effect of FEN1 down-regulation, in vivo, our results showed that FEN1 knockdown inhibited the tumor volume significantly (Fig. 3a–c), and there was no statistical difference in the weight of the three groups (Fig. 3d). Compared with the experimental mice, tumors grew faster in the controls (Fig. 3b). From day 13 to day 28, the tumor volume after FEN1 knockdown was significantly smaller than that in the controls, and the tumor proliferation was not obvious in the whole process (Fig. 3c). Protein-Protein Interaction Networks (PPI) demonstrated that FEN1 have a complex relationship with other proteins associated with DNA damage repair (Supplementary Fig. 2a), then we divided all the samples into FEN1-low and FEN1-high groups according to the expression of FEN1 (Supplementary Fig. 2b), the heatmap showed that the expression of proliferating cell nuclear antigen (PCNA) increased in FEN1-high group. Furthermore, it was showed that FEN1 have a positive correlation with PCNA by Supplementary Fig. 2c. Then, the tumor was dissected and IHC was performed, the results showed that the volume of cell nuclei in controls significantly increased, and PCNA was expressed in most cells in controls and was significantly higher than that in FEN1-shRNA group (Fig. 3e).

By conducting comet assay (Fig. 3f), we found that the Tail DNA proportion of Cal-27 cells in FEN1-shRNA group increased compared with the blank group and the control group. The immunofluorescence (IF) showed that compared with the blank group and NC-shRNA group, the expression of phospho-H2AX (pH2AX) and P53-binding protein 1 (53BP1) of Cal-27 cells in FEN1-shRNA group increased significantly (Fig. 3g, h).

### Down-regulation of FEN1 mediated the antitumor effect of PD-L1 and HLA-DR in OSCC

**Fig. 4:**
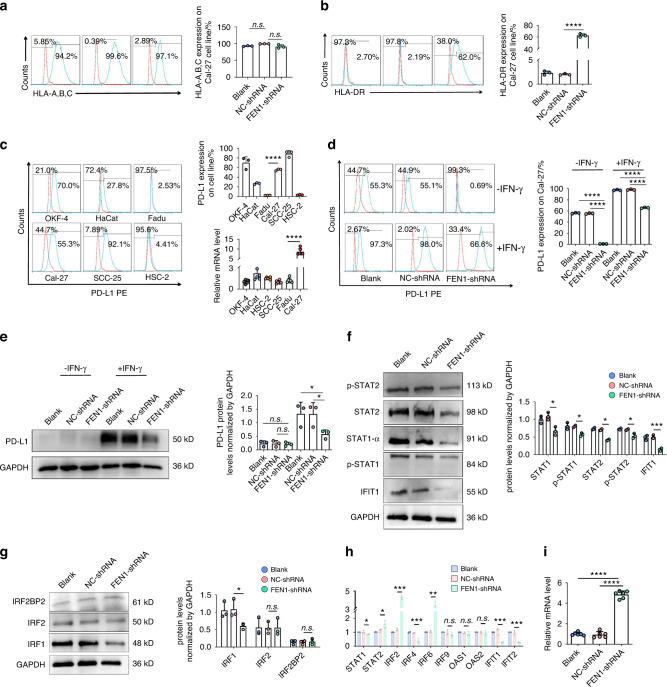
Down-regulation of FEN1 mediated the antitumor effect of PD-L1 and HLA-DR in OSCC. **a** Flow cytometry was performed to verify the expression of HLA-A, HLA-B and HLA-C proteins on the surface of Cal-27 cells in all groups, and the differences were not statistically significant (*n* = 3). **b** The expression of HLA-DR protein on the surface of Cal-27 was validated via flow cytometry, the expression of HLA-DR increased in FEN1-shRNA compared with controls (*n* = 3). **c** Flow cytometry (*n* = 3) and qRT-PCR (*n* = 6) were performed to verify the expression of PD-L1 in different OSCC lineages. **d** Flow cytometry was used to detect the expression of PD-L1 on Cal-27 cells. FEN1 downregulating could effectively inhibit the induction of PD-L1 on Cal-27 cells (*n* = 3). **e** At the protein level, FEN1 downregulation could inhibit the induction of IFN-γ on total protein expression of PD-L1 in Cal-27 (*n* = 3). **f** Western blot was performed to verify that FEN1 knockdown could suppress the expression of p-STAT1、STAT1-α、STAT2、p-STAT2 and IFIT1 in Cal-27 cells (*n* = 3). **g** FEN1 knockdown effectively suppress the expression of IRF1 compared with other groups by western blot (*n* = 3). **h** qRT-PCR were used to show that FEN1 silencing could upregulate the expression of STAT2, IRF2, IRF6, inhibiting the expression of STAT1, IRF4, IFIT1 and IFIT4; and have no influence on the expression of IRF9, OAS1, OAS2 (*n* = 3). **i** qRT-PCR showed that FEN1 knockdown could significantly increase the expression of CIITA (*n* = 6). error bars, mean ± SD; n.s., not significant; *P < 0.05, **P < 0.01, ***P < 0.001; ****P < 0.000 1; by *t* test or two-way ANOVA

A correlation expression analysis showed that FEN1, PD-L1 and HLA-DR were positively correlated with JAK/STAT1 (Supplementary Fig. 3). Performing flow cytometry, we found that HLA-A, HLA-B, and HLA-C were positively expressed in the three groups and that the differences in expression among the groups were not statistically significant (Fig. 4a). HLA-DR was no significantly expressed in the blank group or the NC-shRNA group, but the downregulation of the FEN1 gene induced HLA-DR expression, and the difference compared with the controls was statistically significant (Fig. 4b). In addition, we compared the expression of PD-L1 on the surface of OSCC cell and found that the PD-L1 expression on the surface of OKF-4, Cal-27 and SCC-25 cells was higher than that on the cells of other lineages. qRT-PCR showed that the mRNA level of PD-L1 was the highest in Cal-27 cells (Fig. 4c).

Furthermore, we explored the expression of PD-L1 on the surface of Cal-27 cells by knocking down FEN1. Flow cytometry showed that the expression of PD-L1 in control cell group was much higher than that in the FEN1-shRNA group. Considering these data, we simulated a tumor microenvironment by adding IFN-γ to the cultures. IFN-γ effectively induced the expression of PD-L1 in the blank and NC-shRNA groups, and FEN1 gene downregulation effectively inhibited the induction of PD-L1 by IFN-γ (Fig. 4d). At the protein level, only a small amount of total PD-L1 was expressed in Cal-27 cells without IFN-γ stimulation, and FEN1 downregulation exerted no significant effect on the total PD-L1 expression level (Fig. 4e). Then, the total protein expression of PD-L1 in controls was induced by IFN-γ, and downregulating the expression of FEN1 effectively inhibited the induction of IFN-γ on total PD-L1 protein in Cal-27 cells.

To address the role played by JAK-STAT1 in the regulation of PD-L1 and HLA-DR expression, we performed western blotting (WB) to measure the expression of three DNA-damage-tolerance-related proteins, namely, STAT1, STAT2, and interferon-induced protein with tetratricopeptide repeats 1 (IFIT1). The results showed that the protein levels in the FEN1-shRNA group were significantly decreased compared with those in the control group (Fig. 4f). Moreover, we explored the downstream targets of STAT1 to confirm the hypothesized mechanism. At the protein level, interferon regulatory factor 1 (IRF1) was decreased in the FEN1-shRNA group, however, the expression of IRF2 and IFN regulatory factor 2 binding protein 2 (IRF2BP2) was not different among the groups (Fig. 4g). At the mRNA level, the expression of ISGs was measured in each group, and the results are shown in Fig. 4h. The mRNA expression of STAT1, IRF4 and IFIT1 in the FEN1-shRNA group significantly was downregulated compared with that in the other groups, while the expression of STAT2, IRF2 and IRF6 was upregulated, and the expression of IRF9 and 2’-5’-oligoadenylate synthetase 1 (OAS1) showed no statistically significant difference. Moreover, the mRNA expression of class II major histocompatibility complex transactivator (CIITA) was measured, and the results demonstrated that it was significantly upregulated in the FEN1-shRNA group compared with the other groups (Fig. 4i).

### Immune-related cell surface molecules correlates with the malignancy in OSCC

**Fig. 5:**
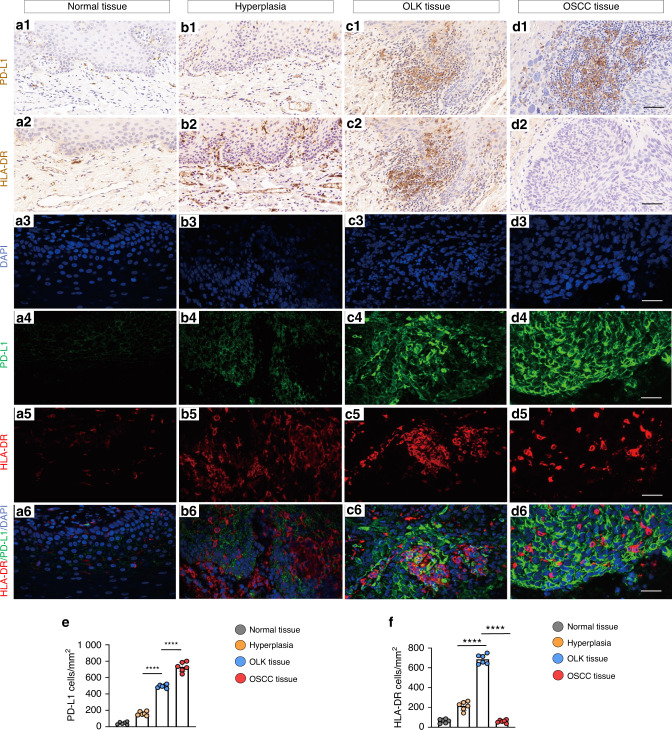
The correlation between the immune-related cell surface molecules and the malignancy in OSCC. **a1**–**d1** PD-L1 staining on normal tissue, OLK and OSCC tissue (*n* = 6). **a2**–**d2** HLA-DR staining on normal tissue, OLK and OSCC tissue (*n* = 6). Scale bar: 100 μm. **a3**–**d3** Representative image of DAPI staining in OLK and OSCC. **a4**–**d4** Representative image of PD-L1 staining in OLK and OSCC. **a5**–**d5** Representative image of HLA-DR staining in OLK and OSCC. Channels were separately presented for a clearer view. **a6**–**d6** Co-immunostaining of HLA-DR and PD-L1 in OLK and OSCC. Scale bar: 200 μm. **e**, **f** Quantification of PD-L1 and HLA-DR expression level in normal, OLK and OSCC tissue. *n* = 6; error bars, mean ± SD, ****P < 0.000 1; *t* test or two-way ANOVA

To verify the relationship between immune-related cell surface molecules and the malignancy of the tissues obtained from the clinic, we performed IHC, and typical IHC staining images are shown in Fig. 5. The expression of programmed death 1 (PD-1), PD-L1 and HLA-DR was higher in OLK group than in the hyperplasia samples (Fig. 5b1-b2, Fig. 5c1-c2, Supplementary Fig. 4). Notably, in the OSCC tissues, the expression of PD-L1 was high, while the HLA-DR staining remained low(Fig. 5d1–d2).

**Fig. 6:**
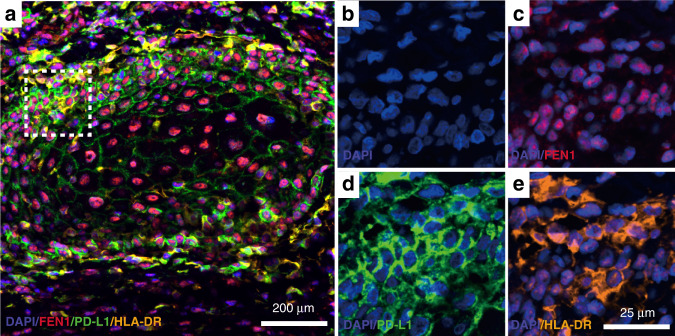
Representative image of multiplex immunohistochemistry. **a** Co-immunostaining representative markers in OSCC. The boxed area was magnified and displayed on the right panels. Scale bar: 200 μm. **b** The staining of DAPI in OSCC. **c** Co-immunostaining of DAPI and FEN1 in OSCC. **d** Co-immunostaining of DAPI and PD-L1 in OSCC. **e** Co-immunostaining of DAPI and HLA-DR in OSCC. Scale bar: 25 μm

To observe the immune-related cell surface molecules directly, mIHC was performed, and the results showed that the green fluorescence emitted by stained PD-L1 was densely distributed in the cell membrane, forming a net-like pattern in the OSCC tissues (Fig. 5d4). In addition, the red fluorescence emitted by stained HLA-DR was dispersed in the cell membrane (Fig. 5d5). As shown in the representative image in the Fig. 6 that FEN1 was overexpressed in OSCC tissues. In addition, PD-L1 was highly expressed at the same time that, in a scattered staining pattern, HLA-DR was expressed.

## Discussion

FEN1, a structure-specific endonuclease with multiple functions, plays an important role in DNA replication and repair.^13^ It is involved in a variety of biological functions, including Okazaki fragment maturation, base excision repair (BER), and trinucleotide repeats.^22^ Thus, FEN1 is a central component in DNA replication and repair,^11,13^ and its defects can lead to genomic instability and cancer susceptibility. Shen et al. established the first batch of FEN1 E160D-mutant mice, which showed signs of autoimmunity-induced chronic inflammation.^15^ FEN1 E160D-mutant mice have also been shown to exhibit higher tumor susceptibility,^23^ indicating that FEN1 may be considered a tumor suppressor gene. However, multiple subsequent studies have found that FEN1 was overexpressed in a variety of solid tumors, and therefore, it was regarded as a marker of poor prognosis.^24–28^ This evidence suggests two mechanisms by which FEN1 promotes the initiation and progression of tumors: genomic instability caused by FEN1 mutation that leads to tumorigenesis and overexpression of FEN1 that promotes the proliferation of tumor cells.

Inhibition of FEN1 expression not only suppressed the proliferation of cancer cells but promotes apoptosis and reduced the drug resistance of cancer cells.^16,25,29–32^ Similarly, in the present study, we showed that the tumor volume was limited by FEN1 downregulation and that FEN1 knockdown significantly attenuated the proliferation and migration of Cal-27 cells, promoted apoptosis and caused cell cycle arrest in the G0/G1 phase. These results indicated that FEN1 downregulation inhibited the growth of OSCC tumors through its effects on various biological behaviors. In addition, as one of the hallmarks of cancers, DNA repair deficiency is an important cause of genomic instability.^33^ Bielinsky et al. revealed that FEN1 drove DNA hypersensitivity in a PCNA-dependent manner.^34^ A mouse model carrying an FEN1 mutation that eliminated the FEN1-PCNA interaction was reported in a study, and the DNA breaks caused by this mutation promoted the development of aneuploid cancers.^14^ Given the critical role that FEN1 plays in DNA damage repair, we conducted DNA damage-related experiments after downregulating FEN1. As expected, the result showed that FEN1 was positively correlated with PCNA and that the DNA in Cal-27 cells was damaged by knocking down FEN1, which explained the inhibitory effect of FEN1 downregulation in OSCC from the perspective of DNA damage repair.

With the development of tumor immunology, our understanding of the immune system in the initiation and progression of tumors is expanding. Genomic instability, cell stress, metabolic changes, apoptosis and disruption to circulatory pathways are all considered to be drivers of malignant processes.^33^ Recent studies have suggested that genomic instability can lead to high levels of mutation and neoantigen loading in tumors, which lead to upregulated PD-L1 expression in tumor microenvironment (TME) cells.^35^ Defects in DNA repair mechanisms have also been associated with prolonged survival and lasting clinical benefits via immune checkpoint blockades (ICBs).^36^ According to the immune-editing hypothesis, tumor cells show downregulated HLA/MHC antigen expression and/or activated immunosuppressive mechanisms during immune editing, for example, the expression of PD-L1, PD-L2, interleukin-6 (IL-6), IL-10 and other immunosuppressive signaling molecules is upregulated, generating a series of malignant immunophenotypes and promoting immune escape.^36,37^ Therefore, avoiding immune destruction becomes one of the hallmarks of cancer.^33^ According to the previously reported portion of our study, we found that FEN1 knockdown caused DNA damage in Cal-27 cells. Moreover, the results of IHC showed that immune cell infiltration was notably in the OSCC microenvironment. Moreover, the infiltration of lymphocytes increased as the degree of malignancy increased, and Foxp3^+^ expression was significantly higher in the low-differentiation OSCC group than in the other groups, suggesting that an inhibitory immune microenvironment may have been established. Furthermore, the trend in FEN1 expression was the same as that described above. In summary, these data indicate that FEN1 might affect OSCC immunity through DNA damage repair.

Thus, we further investigated the possible effects of FEN1 knockdown on the acquisition of two important phenotypes of immune escape. In the current study, the expression of HLA-DR increased with FEN1 downregulation, which indicated that FEN1 downregulation could induce the expression of Cal-27 cells surface antigen. Multiple studies have noted that compared with normal cells, HLA/MHC, as one of the most important phenotypic changes in the immune escape process of tumor cells, is downregulated or absent in a variety of tumor cells and is closely related to the poor prognosis of patients.^38^ Our data also proved that the expression of HLA-DR decreased in OSCC (Fig. 5), suggesting that downregulating FEN1 can improve the immunogenicity in OSCC. In addition, in the present study, we found that FEN1 downregulation can inhibit the expression of PD-L1. Studies have shown that the combination of PD-1 and PD-L1 can suppress the immune activity of T cells by directly inhibiting the proliferation and secretion of T cells, promoting apoptosis, and suppressing the migration and differentiation of T cells.^39,40^ The upregulated expression of PD-L1 in tumor cells may mediate immune escape of cancer cells. PD-L1 is highly expressed in various tumor cells, but it is not expressed or expressed at low levels in normal tissues.^41–43^ In HNSCC, the expression of PD-L1 has been correlated with low overall and disease-free survival and high lymph node metastasis.^44^ Importantly, PD-L1 is considered to be an independent poor prognosis marker in OSCC.^45^ Similarly, in this study, we provided data showing that the expression of PD-L1 was mostly high in OSCC (Fig. 5) and that FEN1 downregulation can effectively inhibited the expression of PD-L1 in Cal-27 cells, indicating that FEN1 downregulation may relieve the inhibition of T cells and improve the immune activity in OSCC.

**Fig. 7:**
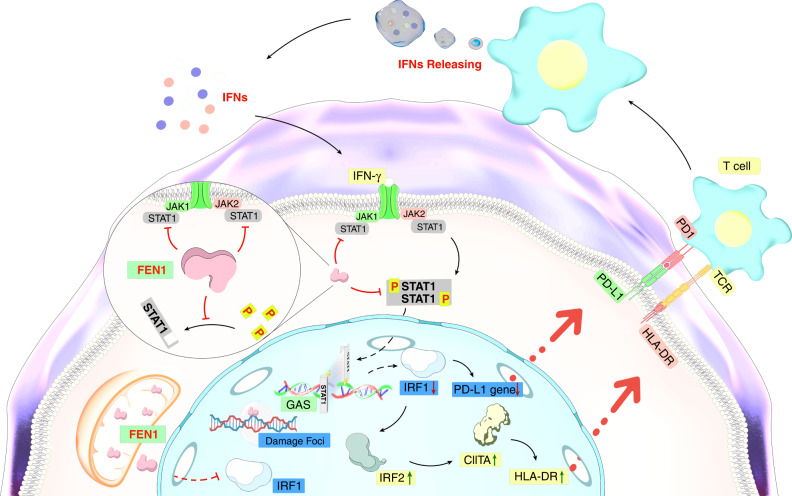
Schematic overview of FEN1 downregulation affects PD-L1 and HLA-DR via IFN-γ/JAK/STAT1 pathway in Cal-27 cells. TYK2, tyrosine kinase 2; TCR, T cell receptor

IFN-γ, a pleiotropic cytokine, plays a critical role in immunomodulatory effects.^46^ The antitumor function of IFN-γ includes regulating antigen presentation, and promoting inflammatory and chemotactic signals.^47^ The biological effects of IFN-γ are elicited mainly through the canonical JAK/STAT signaling pathway.^48^ Recent work has also shown several noncanonical IFN-γ-activated pathways, for example, Gao et al. proposed that IFN-γ suppressed the expression of PD-L1 by activating the JAK/STAT and Phosphatidylinositol-3-kinase (PI3K)/Akt pathways in adenocarcinoma.^49^ Another study showed that IFN-γ activated the PI3K/Akt/mammalian target of rapamycin (mTOR) pathway in lung epithelial cells. However, to determine whether this noncanonical regulation of IFN-γ is independent of JAK/STAT signaling, further exploration is needed.^50^ The JAK/STAT pathway is part of a signaling cascade consisting of several ligands, tyrosine kinase-related receptors and the STAT family of proteins.^51^ IFN-γ binds to its receptors, interferon gamma receptor 1 (IFNGR1) and IFNGR2. This binding activates JAKs and provides a docking site for STAT1.^52^ STAT1 is phosphorylated after binding to activated receptors through its SH2 domain and then forms homologous dimers that enter the nucleus and bind to gamma-interferon activation sites (GAS). Then, the transcription and expression of IFN-γ inducer genes (e. g. IRF1) are upregulated.^53^ In this study, we used IFN-γ to simulate the tumor microenvironment, and the results showed that FEN1 downregulation effectively inhibited the expression of PD-L1 that had been induced by IFN-γ, indicating that FEN1 downregulation may regulate the expression of PD-L1 through the JAK/STAT signaling pathway and IFN-γ induction (such as IRF1) (Fig. 7).

Several studies have revealed that, as a downstream mediator in the IFN-γ/JAK/STAT1 pathway, the absence of IRF1 inhibited tumor progression and was necessary for the regulation of PD-L1.^54^ It exerted antitumor effects on IFN-γ by inducing proteasome subunits, antigen-processing-related transporters, and MHC expression and by increasing cytokine secretion.^46^ In addition, as members of the IRF family, IRF1 and IRF2 show antagonistic effects on each other, with IRF1 inhibiting the function of IRF2.^55^ Studies have shown that IRF2 upregulated the expression of CIITA, which played a pivotal role in promoting the expression of MHC II (namely HLA-DR).^56^ In the present study, FEN1 knockdown suppressed the expression of both STAT1 and IRF1 at the protein level. To further elucidate the possible mechanism mediating the effect of FEN1 downregulation on PD-L1, WB was performed to explore the effect of FEN1 knockdown on the expression of IRF2 and IRF2BP2, and the results demonstrated that FEN1 exerted no significant effect on these two proteins. To observe the effect of FEN1 downregulation on ISGs expression in Cal-27 cells, we performed qRT-PCR and the results demonstrated that the expression of STAT2, IRF2 and IRF6 increased. We further examined the effect of FEN1 downregulation on the expression of CIITA and found that the mRNA level in the FEN1-shRNA group was significantly increased compared with that in the control group. These data provide evidence showing that FEN1 downregulation might inhibit the expression of IRF1 in the nucleus through the JAK/STAT1 pathway, and further upregulates the expression of IRF2 and CIITA, eventually regulating the expression of PD-L1 and HLA-DR, respectively. However, the regulatory mechanism of PD-L1 and HLA/MHC is quite complex, and to determine whether the regulatory mechanism related to FEN1 downregulation is JAK-STAT1-dependent, further study is needed.

FEN1 is a core protein in DNA damage repair, and this study describes a novel function of FEN1; that is, FEN1 exerts an immunomodulatory effect by regulating the expression of PD-L1 and HLA-DR through the IFN-γ/JAK/STAT1 pathway. IRF1 is a target gene downstream of the IFN-γ/JAK/STAT1 pathway, and its deficiency can reduce tumor progression. IRF1 competitively inhibits the IRF2, which enables us to regulate two immunophenotypes at the same time by knocking down FEN1. Our study reinterprets FEN1 behavior from the perspective of immunotherapy, providing theoretical evidence that FEN1 may be a potential immunotherapy target in OSCC.

## Materials and methods

### Patients and clinical tissue collection

All samples were obtained from the participants who underwent treatments in the West China Hospital of Stomatology at Sichuan University during 2015–2019. In total, 91 OSCC patients, 28 OLK patients and 13 patients who received third molar extraction as normal controls were enrolled in our study. None of the patients received chemoradiotherapy or other biological therapy before the biopsy or extraction. This work was approved by the Human Research Ethics Committee of West China Hospital of Stomatology at Sichuan University (WCHSIRB-D-2015-111).

### Histology and immunohistochemistry

The paraffin-embedded tissues were cut into 4-μm thick sections used for Hematoxylin-eosin (H&E) staining or immunostaining procedures. HE staining was used for histological examination and the following steps were performed according to standard histology protocol. For IHC, primary antibodies were applied and incubated at 4 °C overnight. A Diaminobenzidine (DAB) detective kit (ZLI-9018, ZSGB-BIO, Beijing, China) was used. Antibodies against FEN1, CD3, CD4, CD8, Foxp3, PD-1, PD-L1 and HLA-DR were used. Detailed information is provided in Supplementary Table 3. Hematoxylin was used for nuclear staining, and neutral gum was used to cover the slides. Specific antigens in ten randomly-selected fields were visualized by two independent investigators using a light microscope at a power of ×400.

### Cell line culture and transfection

The human OSCC cell lines Cal-27, OKF-4, HaCat, Fadu, SCC-25 and HSC-2 were obtained from State Key Laboratory of Oral Diseases or Frontier Innovation Center for Dental Medicine Plus. Cells were maintained in 37 °C incubator with 5% CO_2_. Cal-27 cells (5 × 10^3^ per well in 100 μL) were cultivated in 96-well plates and grown to 50% 18–24 h at 37 °C overnight before lentivirus transfection. Prepare the mixture of ENi.S. liquid and polybrene to make sure the working concentration (5 μg·mL^−1^). The original medium was removed and ENi.s containing 5 μg/ml polybrene was added (100 μL per well), different MOI values were set (0, 30, 40, 50, 60, 80, 100), and according to the formula: required amount of disease venom (ml) = MOI x N/lentivirus titer, appropriate amount of LV-Control virus suspension was added, incubated at 37 °C for 8–12 h, and then the medium containing virus was replaced with fresh medium. After 72 h, transfection efficiency and MOI were determined according to the fluorescence expression of each group. Transfection efficiency = (number of cells emitting green fluorescence/total number of cells under visible light) x 100%. 72 h after transfection, qRT-PCR and WB were performed to verify the interference efficiency. The sequences used for targeting genes are listed in supplementary table 5.

### Establishment of xenograft mouse models

In total, 4–6 weeks old BALA/c nude mice (21 females) were purchased from the State Key Laboratory of Biotherapy and Cancer Center at Sichuan University. Mice were randomly divided into 3 groups. All animal experiments were performed following protocols approved by the U.S. Public Health Service’s policy on the humane care and use of laboratory animals. Tumor cells (5 × 10^6^) were suspended in 40 μl with 50% PBS and 50% matrigel, then injected into the flanks of nude mice to form xenografts. All experimental protocols were approved by the Research Ethics Committee of West China Hospital of Stomatology, Sichuan University (WCHSIRB-D-2022-037).

### Western blot analysis

Total protein extracts were obtained from cells utilizing total protein extraction kit (PE001, SAB, Maryland, USA). Proteins in the nucleus were extracted using nuclear and cytoplasmic protein extraction kit (P0027, Beyotime, Shanghai, China). The concentration of proteins was quantified using the bicinchoninic acid (BCA) protein assay kit (CW0014S, CWBIO, Jiangsu, China). After denaturation by sodium dodecyl sulfatepolyacrylamide gel electrophoresis (SDS-PAGE) loading buffer, proteins were separated via 10% SDS-PAGE and transferred onto 0.45 μm polyvinylidene fluoride (PVDF) membranes. The membranes were then blocked with 5% bovine serum albumin (BSA; A1933, Sigma-Aldrich) for 1 h at room temperature and incubated overnight with primary antibodies (Supplementary Table 3) at 4 ^◦^C. On the following day, the membranes were washed 3 times with TBST before re-probed with goat anti-rabbit antibody (Supplementary Table 3) for 1 h at room temperature. Next, the membranes were exposed to a Gel imager system (BioSpectrum®600, UVP, USA) to detect reactive signals after incubation with Immobilon western chemiluminescent HRP substrate reagent (WBKLS0500, Millipore, USA). Image J software was used to quantify the band intensity. Relative protein expression levels of the target proteins were normalized against the expression level of GAPDH.

### Quantitative real-time PCR (qRT-PCR)

Total RNA was isolated using TRIzol (Invitrogen, Thermo Fisher Scientific Inc., California, USA), the primer sequences used in this study were shown in Supplementary Table 4. GAPDH was used as the endogenous control. All the procedures were performed according to the manufacturer’s instructions. Additionally, the comparative CT method (ΔΔCT method) was used to quantify target gene expression in comparison with the control.

### Flow cytometry

In total, 2 × 10^6^ cells (per group) were harvested and washed repeatedly with PBS for three times, cells were re-suspended in 5 mL of cell staining buffer. Centrifuging for 5 min (300 g) and discarding the supernatant. Then, each group of cells was re-suspended in 800–1 200 μL cell staining buffer and divided in blank group, compensation group and experiment group (some of them need to be interfered with IFN-γ before staining). Appropriate amount of flow cytometry antibody and corresponding homologous control antibody were added, and then incubated for 20–30 min in the ice box. Detailed information of the antibodies was showed in supplementary table 3. Cells were re-suspended in 2 ml of cell staining buffer and washed 2 times. All the groups were detected by flow cytometry (CytoFLEX, Beckman coulter, USA) and analyzed by CytExpert software.

### Data collection and bioinformatic analysis

The OSCC-related dataset (GSE30784) was downloaded from gene expression omnibus (GEO) database (http://www.ncbi.nlm.nih.gov/geo/). The PPI was generated by STRING database (https://string-db.org/). Correlation expression analysis was performed by Rstudio through the packages named limma, ggplot2, ggpubr and ggExtra.

### Multiplex immunohistochemistry staining

For the mIHC staining in this study, primary antibodies (detailed information showed in supplementary Table 3) as well as a multicolor-kit (Absin, China) were used. We used Opal 520 channel for PD-L1 [fluorescein isothiocyanate (FITC), a green fluorescence stain], Opal 570 channel and Opal 670 for HLA-DR [cyanine 3 (Cy3), an orange fluorescence stain for Fig. 6. cyanine 5 (Cy5), a red fluorescence stain for Fig. 5.], Opal 670 channel for FEN1 [cyanine 5 (Cy5), a red fluorescence stain], and DAPI (4′,6-diamidino-2-phenylindole, a blue fluorescence stain). All the 20 slides were observed and imaged by Olympus FV1200 confocal system (Tokyo, Japan). All the images were analyzed by ImageJ software (NIH, Bethesda, MD, USA).

### Other methods

Colony formation assay, cell viability assay, cell apoptosis, cell cycle analysis, immunofluorescence and comet assay were described in details in supplemental information.

### Statistical analysis

GraphPad Prism 8 software (GraphPad) was used for all statistical analysis. Data were analyzed using a *t* test (if not indicated otherwise) and presented as the means ± SD. *P* values were considered statistically significant at <0.05.

## Supplementary information

Supplementary Information

## Data Availability

The data presented in this study are included in all the figures and supplementary materials, which could be provided from the corresponding author after reasonable demand.
