# Supplementary material for: Down-regulation of DNA key protein-FEN1 inhibits OSCC growth by affecting immunosuppressive phenotypes via IFN-γ/JAK/STAT-1
Source: Int J Oral Sci. 2023 Apr 25;15:17. doi: 10.1038/s41368-023-00221-8 (PMC10130046; doi:10.1038/s41368-023-00221-8)
Supplement: Supplementary file 1 — Supplementary Information [file 41368_2023_221_MOESM1_ESM.docx]

***Supplementary Information***

***Supplementary Tables***

**Supplementary Table 1.**

Correlation between FEN1 expression and clinicopathologic features of oral leukoplakia

| **Parameters** | | **Number**  **(n=28)** | **Positive cell rate (%)** | **Staining power** | **Staining score** | ***P value*** |
| --- | --- | --- | --- | --- | --- | --- |
| **Gender** | **Male**  **Female** | 17 | 1.35±0.60 | 2.18±0.53 | 2.59±1.00 | 0.193 |
|  |  | 11 | 1.91±1.04 | 1.91±0.54 | 2.82±1.17 |  |
| **Age** | **＜60**  **≥60** | 18 | 1.56±0.92 | 2.06±0.54 | 2.61±1.04 | 0.659 |
|  |  | 10 | 1.60±0.70 | 2.10±0.57 | 2.80±1.14 |  |
| **Position** | **Cheek**  **Tongue**  **Others** | 11 | 1.36±0.67 | 2.00±0.632 | 2.36±1.03 | 0.280 |
|  |  | 16 | 1.69±0.95 | 2.13±0.77 | 2.81±1.05 |  |
|  |  | 1 | 2.00±0.00 | 2.00±0.00 | 4.00±0.00 |  |
| **Classify** | **Hyperplasia**  **Low grade**  **High grade** | 14 | 1.36±0.50 | 1.86±0.54 | 2.29±1.07 | 0.005 |
|  |  | 8 | 1.13±0.35 | 2.25±0.46 | 2.50±0.76 |  |
|  |  | 6 | 2.67±1.03 | 2.33±0.52 | 3.83±0.41 |  |

FEN1 expression was not correlated with gender, age or site of OLK (P>0.05). The expression of FEN1 was positively correlated with the degree of epithelial dysplasia in patients (P=0.005).

**Supplementary Table 2.**

Correlation between FEN1 expression and clinicopathological features of OSCC

| **Parameters** | **Number**  **(n=91)** | | **Positive cell rate**  **(%)** | **Staining power** | **Staining**  **score** | ***P value*** |
| --- | --- | --- | --- | --- | --- | --- |
| **Gender** | **Male** | 56 | 1.89±0.91 | 2.39±0.73 | 3.42.±0.98 | 0.113 |
|  | **Female** | 35 | 2.03±0.71 | 2.40±0.50 | 3.71±0.62 |  |
| **Age**  **(20-80)** | **＜60** | 38 | 1.79±0.88 | 2.39±0.72 | 3.29±1.04 | 0.035 |
|  | **≥60** | 53 | 2.06±0.80 | 2.40±0.60 | 3.68±0.70 |  |
| **Position** | **Cheek** | 33 | 1.82±0.77 | 2.21±0.82 | 3.39±1.06 | 0.056 |
|  | **Tongue** | 47 | 1.84±0.87 | 2.49±0.51 | 3.51±0.80 |  |
|  | **Mouth floor** | 5 | 2.60±0.77 | 2.80±0.45 | 3.80±0.45 |  |
|  | **Gingival** | 3 | 2.33±0.77 | 2.33±0.58 | 4.00±0.00 |  |
|  | **Others** | 3 | 2.00±0.00 | 2.33±0.58 | 4.00±0.00 |  |
| **Dfferentiated degree** | **High** | 56 | 1.68±0.86 | 2.27±0.65 | 3.125±1.03 | 0.008 |
|  | **Medium** | 25 | 1.92.±0.81 | 2.52±0.71 | 3.60±0.82 |  |
|  | **Low** | 10 | 2.70±0.67 | 2.80±0.42 | 4.00±0.00 |  |
| **Tumor size** | **≤4cm** | 63 | 1.73±0.83 | 2.43±0.61 | 3.27±0.94 | 0.224 |
|  | **＞4cm** | 28 | 2.14±0.93 | 2.32±0.78 | 3.53±1.00 |  |
| **Metastasis** | **N0** | 68 | 1.71±0.81 | 2.40±0.65 | 3.22±0.99 | 0.024 |
|  | **N1 or N2** | 23 | 2.30±0.93 | 2.39±0.72 | 3.74±1.00 |  |
| **Stages** | **I** | 30 | 1.67±0.71 | 2.23±0.68 | 3.17±1.02 | 0.197 |
|  | **II** | 33 | 1.82±0.92 | 2.61±0.50 | 3.40±0.86 |  |
|  | **III or IV** | 28 | 2.11±0.96 | 2.32±0.77 | 3.60±0.87 |  |

The relationship between FEN1 expression and clinicopathologic indicators (including sex, age, site, degree of differentiation, tumor size, local lymph node metastasis, and clinical stage) was evaluated.

**Supplementary Table 3.**

Antibodies used in the current study

| **Antibodies** | **Cat No.** | **Dilutions** | **Brand** |
| --- | --- | --- | --- |
| **Immunohistochemistry** |  |  |  |
| Anti-FEN1 | ab133311 | 1:1000 | Abcam |
| Anti-CD3 | HA720082 | 1:600 | HUABIO |
| Anti-CD4 | ET1609-52 | 1:200 | HUABIO |
| Anti-CD8 | ET1606-31 | 1:200 | HUABIO |
| Anti-Foxp3 | ab20034 | 1:500 | Abcam |
| Anti-PD-1 | ET1606-41 | 1:200 | HUABIO |
| Anti-PD-L1 | HA721176 | 1:400 | HUABIO |
| Anti-HLA-DR | ET1610-66 | 1:100 | HUABIO |
| **Western blot** |  |  |  |
| Anti-FEN1 | ab133311 | 1:5000 | Abcam |
| Anti-STAT1α | ab92506 | 1:1000 | Abcam |
| Anti-p-STAT1 | 9167S | 1:1000 | Cell Signaling Technolog |
| Anti-STAT2 | ab32367 | 1:5000 | Abcam |
| Anti-p-STAT2 | 4441T | 1:1000 | Cell Signaling Technolog |
| Anti-IFIT1 | ab305031 | 1:1000 | Abcam |
| Anti-IRF1 | ab243895 | 1:1000 | Abcam |
| Anti-IRF2 | ab124744 | 1:1000 | Abcam |
| Anti-IRF2BP2 | ab217361 | 1:1000 | Abcam |
| Anti-GAPDH | ab9485 | 1:2500 | Abcam |
| Goat anti-Rabbit IgG antibody | HA1012 | 1:5000 | Santa cruz |
| Recombinant Human/Murine IFN-γ | 31505 | 10ng/ml | PeProtech, Inc |
| **Immunofluorescence** |  |  |  |
| Anti-Histone H2A.X | ab124781 | 1:1000 | Abcam |
| Anti-53BP1 | ab175933 | 1:250 | Abcam |
| Goat Anti-Rabbit IgG (H+L) (Alexa-Fluor 488) | #4412 | 1:2000 | Cell Signaling Technology |
| Goat Anti-Rabbit IgG (H+L) (Alexa-Fluor 594) | #8889 | 1:2000 | Cell Signaling Technology |
| DAPI | BL105A | - | Biosharp |
| **Flow cytometry** |  |  |  |
| PE anti-human HLA-A,B,C Antibody | 311405 | 5μl/test | BioLegend |
| PE Mouse IgG2a, κ Isotype Ctrl (FC) Antibody | 400213 | 5μl/test | BioLegend |
| PE anti-human HLA-DR Antibody | 307605 | 5μl/test | BioLegend |
| PE Mouse IgG2a, κ Isotype Ctrl Antibody | 400211 | 5μl/test | BioLegend |
| CD274 (PD-L1, B7-H1)Monoclonal Antibody (MIH1),PE, eBioscience ^TM^ | 12-5983-41 | 5μl/test | Thermo Fisher Scientific |
| Mouse IgG1 κ Isotype Control, PE, eBioscience ^TM^ | 12-4714-41 | 5μl/test | Thermo Fisher Scientific |
| PE anti-mouse CD274 (B7-H1, PD-L1) Antibody | 124307 | 2.5μl/test | BioLegend |
| PE Rat IgG2a, λ Isotype Ctrl Antibody | 402303 | 2.5μl/test | BioLegend |
| **Multiplex Immunohistochemistry** |  |  |  |
| Anti-FEN1 | ab133311 | 1:5000 | Abcam |
| Anti-PD-L1 | HA721176 | 1:400 | HUABIO |
| Anti-HLA-DR | ET1610-66 | 1:100 | HUABIO |

**Supplementary Table 4.**

Primer sequences used in qRT-PCR.

| **Primers** | **Sequence (forward/reverse)** | **Usage** |
| --- | --- | --- |
| *r-Gapdh* | 5’-GAACGGGAAGCTCACTGG-3’  5’-GCCTGCTTCACCACCTTCT-3’ | qRT-PCR |
| *r-Fen1* | 5’-CCAGAAGCACAAGAGCATCGA-3’  5’-CATTTGGCTCGCTCCACTTC -3’ | qRT-PCR |
| *r-Pd-l1* | 5’-GCTATGGTGGTGCCGACTACA-3’  5’-CAGGACTTGATGGTCACTGCTTG -3’ | qRT-PCR |
| *r-Stat1* | 5'-CATTCACATGGGTGGAGCG -3'  5'-GGGTTCAACCGCATGGAAG -3' | qRT-PCR |
| *r-Stat2* | 5’-AAGCACTGCTAGGCCGATTA -3’  5’-GGCTGGGTTTCTACCACAAA -3’ | qRT-PCR |
| *r-Irf2* | 5’-GGAAATAATGCCTTCAGGGTCTAC -3’  5’-AACTGGTTCTTGCTTGATGTGCT -3’ | qRT-PCR |
| *r-Irf4* | 5’-GCGGTGCGCTTTGAACAAG -3’  5’-ACACTTTGTACGGGTCTGAGA -3’ | qRT-PCR |
| *r-Irf6* | 5’-AGAGAAGCAGCCACCGTTTGAG -3’  5’-GATCATCCGAGCCACTACTGGA -3’ | qRT-PCR |
| *r-Irf9* | 5’-GCCCTACAAGGTGTATCAGTTG -3’  5’-TGCTGTCGCTTTGATGGTACT -3’ | qRT-PCR |
| *r-Ifit1* | 5'-TGGGTTCAAGTGGGTTAT-3'  5’-GGCCTTGGCCCGTTCATAAT -3’ | qRT-PCR |
| *r-Ifit2* | 5’-AGCGAAGGTGTGCTTTGAGA -3’  5’-GAGGGTCAATGGCGTTCTGA -3’ | qRT-PCR |
| *r-Oas1* | 5’-GACGCTGACCTGGTTGTCTTCC -3’  5’-GGAGCCTGGACCTCAAACTTCA -3’ | qRT-PCR |
| *r-Oas2* | 5’-ACCCGAACAGTTCCCCCTGGT -3’  5’-ACAAGGGTACCATCGGAGTTGCC -3’ | qRT-PCR |
| *r-Ciita* | 5’-CCTGGAGCTTCTTAACAGCGA-3’  5’-TGTGTCGGGTTCTGAGTAGAG -3’ | qRT-PCR |

***Supplementary Materials and Methods***

**Colony formation assay**

Cells of three groups were inoculated into 24-well plates at a density of 1000 cells per well. After culturing for 14 days, each well of the plates were washed with PBS, dried at room temperature and fixed with 4% formaldehyde for 15 minutes and stained with 0.1% Giemsa for 10 min. Then dried at room temperature. The number of colonies were counted and photographed.

**Cell viability assay**

Lactate dehydrogenase cytoxicity assay kit (LDH, Beyotime, Shanghai, China) and Cell Counting Kit-8 (CCK-8, APE×BIO, USA) were used to determine the cell proliferation together. Both assays were performed according to the manufacturer’s instructions. The OD values were compared and analyzed.

**Cell apoptosis analysis**

Annexin V-FITC/PI apoptosis kit (A11015, PeproTech, China) was used to analyze the cell apoptosis. 2×10^6^ cells (per group) were harvested and washed with pre-cooled PBS, then they were divided in one blank tube, two single stained tubes and three experiment tubes. 5μl Annexin V-FITC and 10 μl PI were added in each experiment tube, two single stained tubes were added either 5μl Annexin V-FITC or 10μl PI, gently blending tubes and incubating 5 min in the dark room. All samples were detected by flow cytometry (CytoFLEX, Beckman coulter, USA) and analyzed by CytExpert software.

**Cell cycle analysis**

Cell cycle staining Kit (A10932, PeproTech, China) was used to determine the cell cycle of the FEN1-shRNA group and the controls. 2 × 10^5^ - 1 × 10^6^ cells were collected and washed with PBS. 1ml DNA staining solution and 10μl permeabilization solution were added in the cell suspension. All the samples were then detected by flow cytometry (CytoFLEX, Beckman coulter, USA) and analyzed by modfit software.

**Immunofluorescence**

Cell crawl tablets were fixed in 4% paraformaldehyde for 20 min and permeated in 0.5% Triton X-100 at room temperature for 20 min. Then, they were blocked with 5% bovine serum albumin (BSA, A1933, Sigma-Aldrich, USA) for 30 min at room temperature min and incubated overnight with primary antibodies (Supplementary Table 3) at 4^◦^C. On the following day, the cell crawl tablets were washed 3 times with PBS before reprobed with goat anti-rabbit IgG (H+L) (Supplementary Table 3) for 1 h at room temperature. DAPI was added and incubated for 5 min, the cell crawl tablets were washed 3 times with PBST (5min × 4). The acquired images were observed under the inverted fluorescence microscope (Olympus IX71, Japan).

**Comet assay**

Cal-27 cells were isolated and prepared as single cell suspension. Preparing the rubber plate according to the DNA damage detection kit (KGA240, KeyGEN BioTECH, Jiangsu, China). After removing the cover glass, the prepared rubber plate was immersed in cell lysis solution chilled at 4^◦^C for 1-3h. Taking out the rubber plate, then rinsing it with double steaming water and putting it in the electrophoresis tank, soakting it in the electrophoresis solution pre-cooled at 4^◦^C for 1h. At the end of electrophoresis, the rubber plate is placed in the neutralizing solution for 30min (10min/time). After drying, staining the rubber plate with PI for 5-10min in the dark room. Rinsing it with distilled water for 10min (5min/time). The acquired images were observed under the inverted fluorescence microscope (Olympus IX71, Japan).

***Supplementary Figure***

***Figure S1.***

***
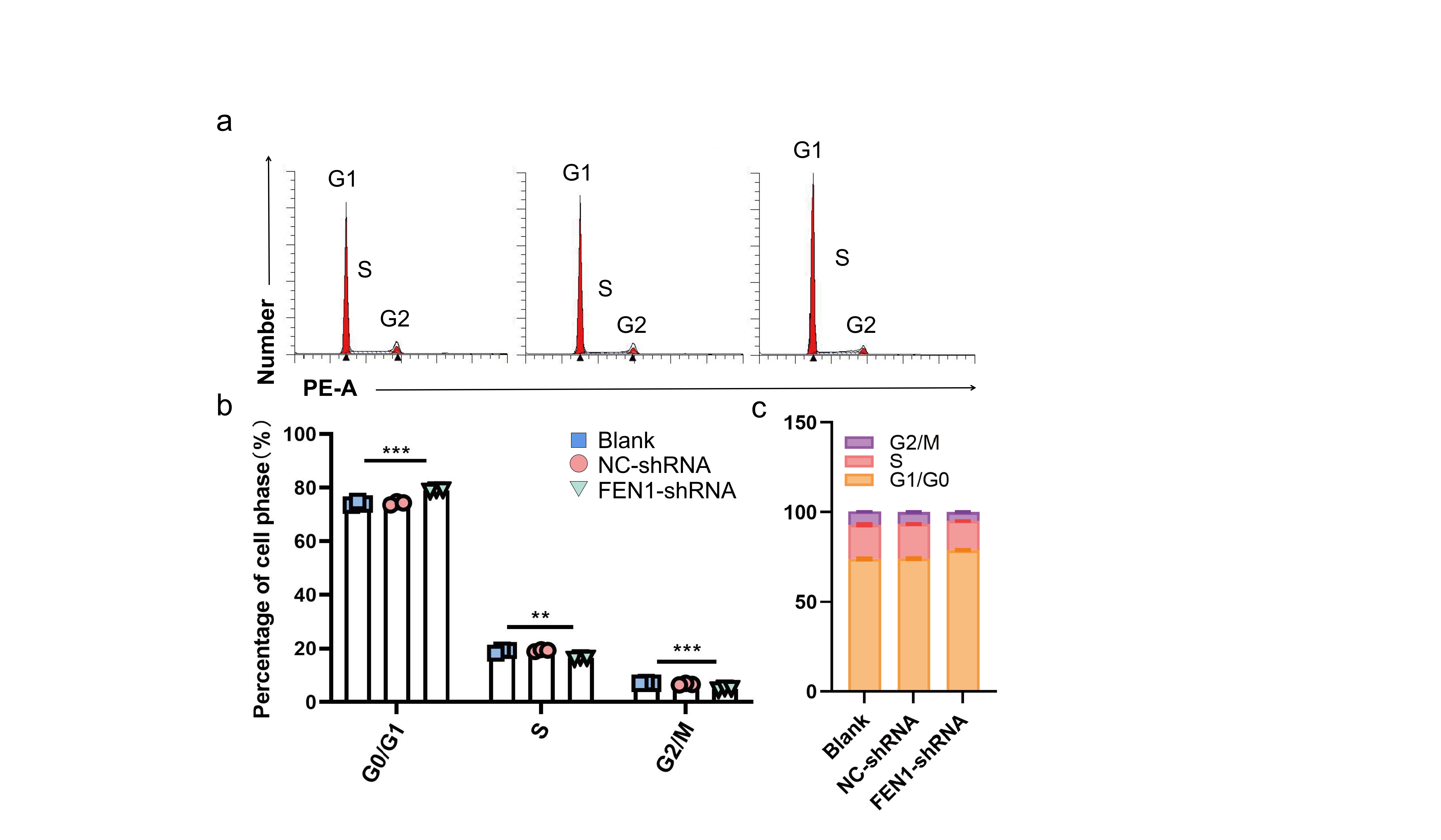
***

**Fig. S1 Cell Cycle Characteristic of FEN1 down-regulation**

***Figure S2.***

***
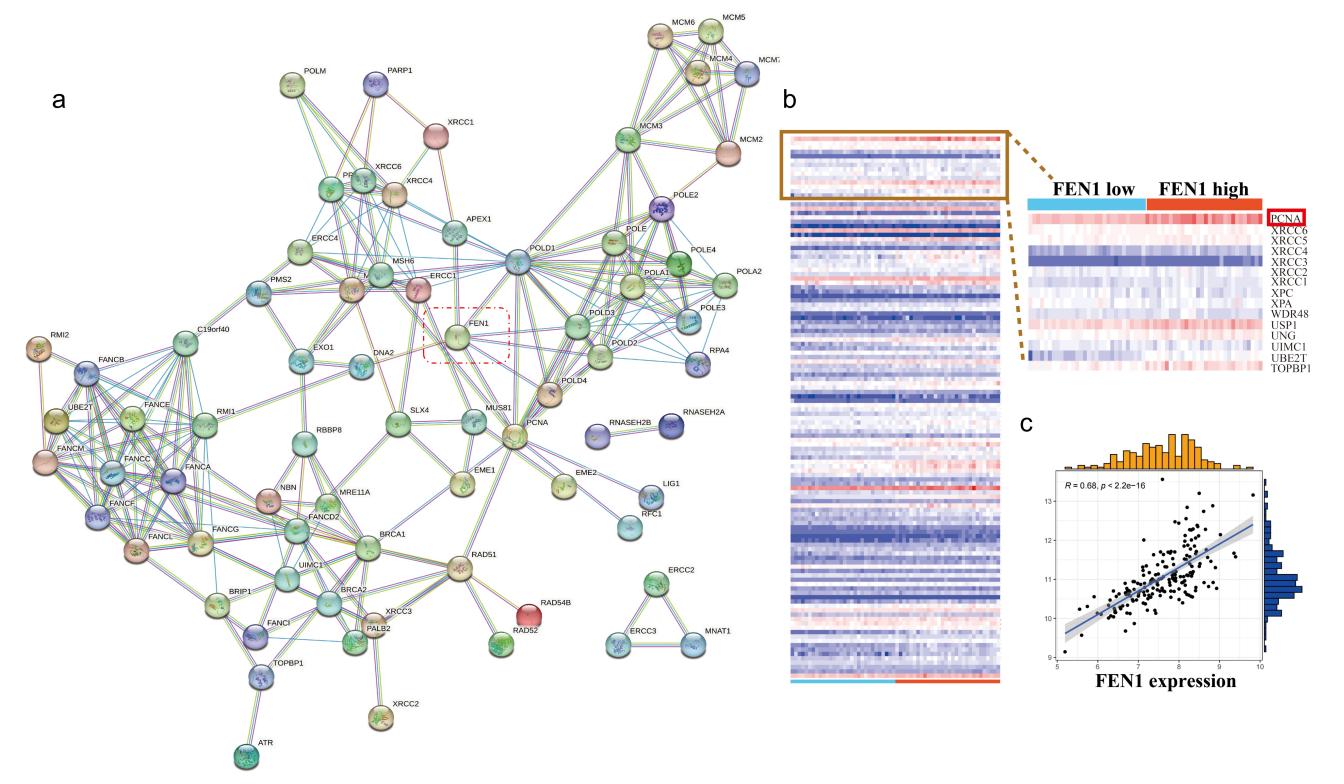
***

**Fig. S2 The relationship of FEN1 and PCNA**

***Figure S3.***

***
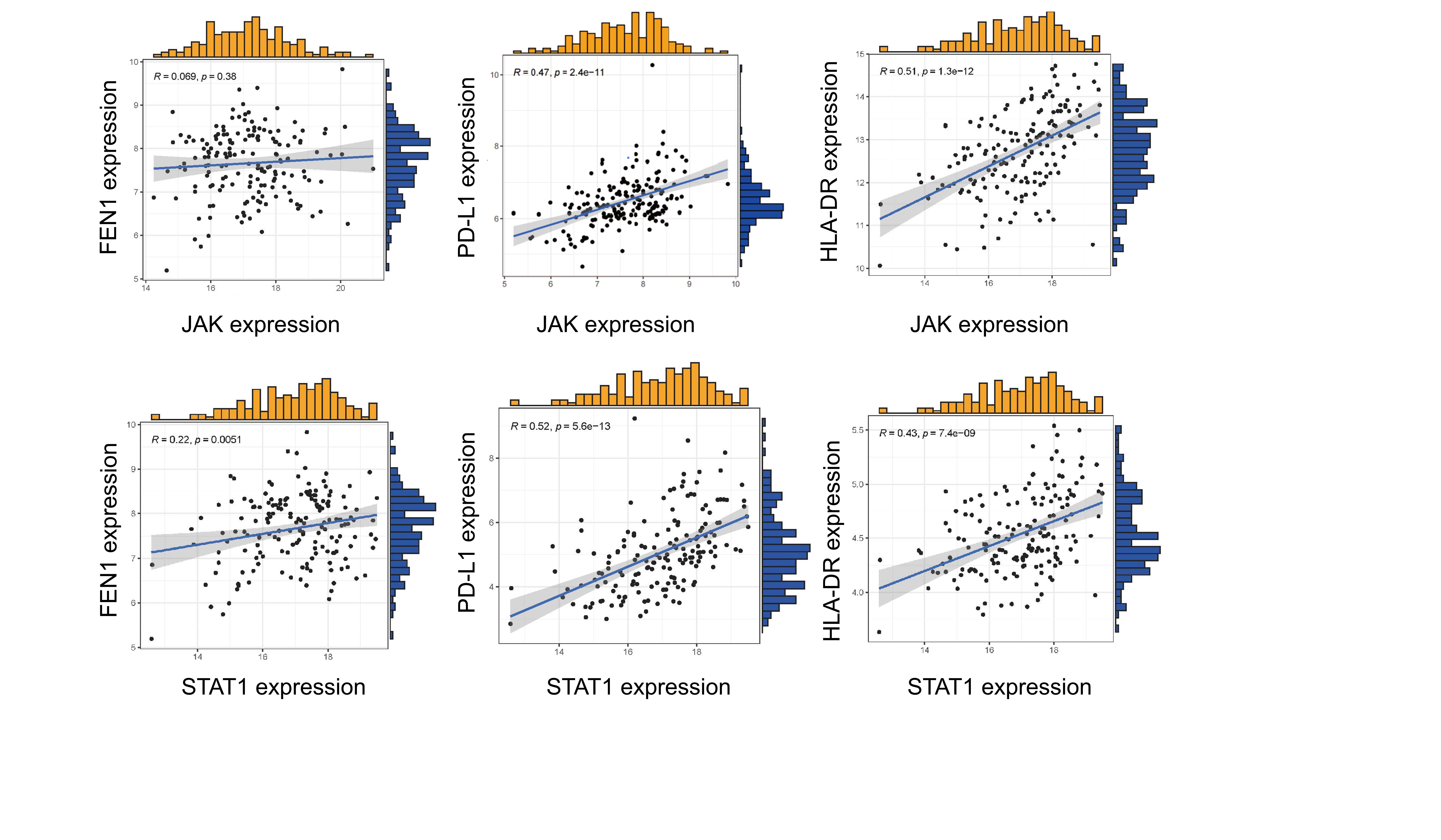
***

**Fig. S3 The correlation of FEN1, PD-L1 and HLA-DR expression with JAK/STAT1**

***Figure S4.***

***
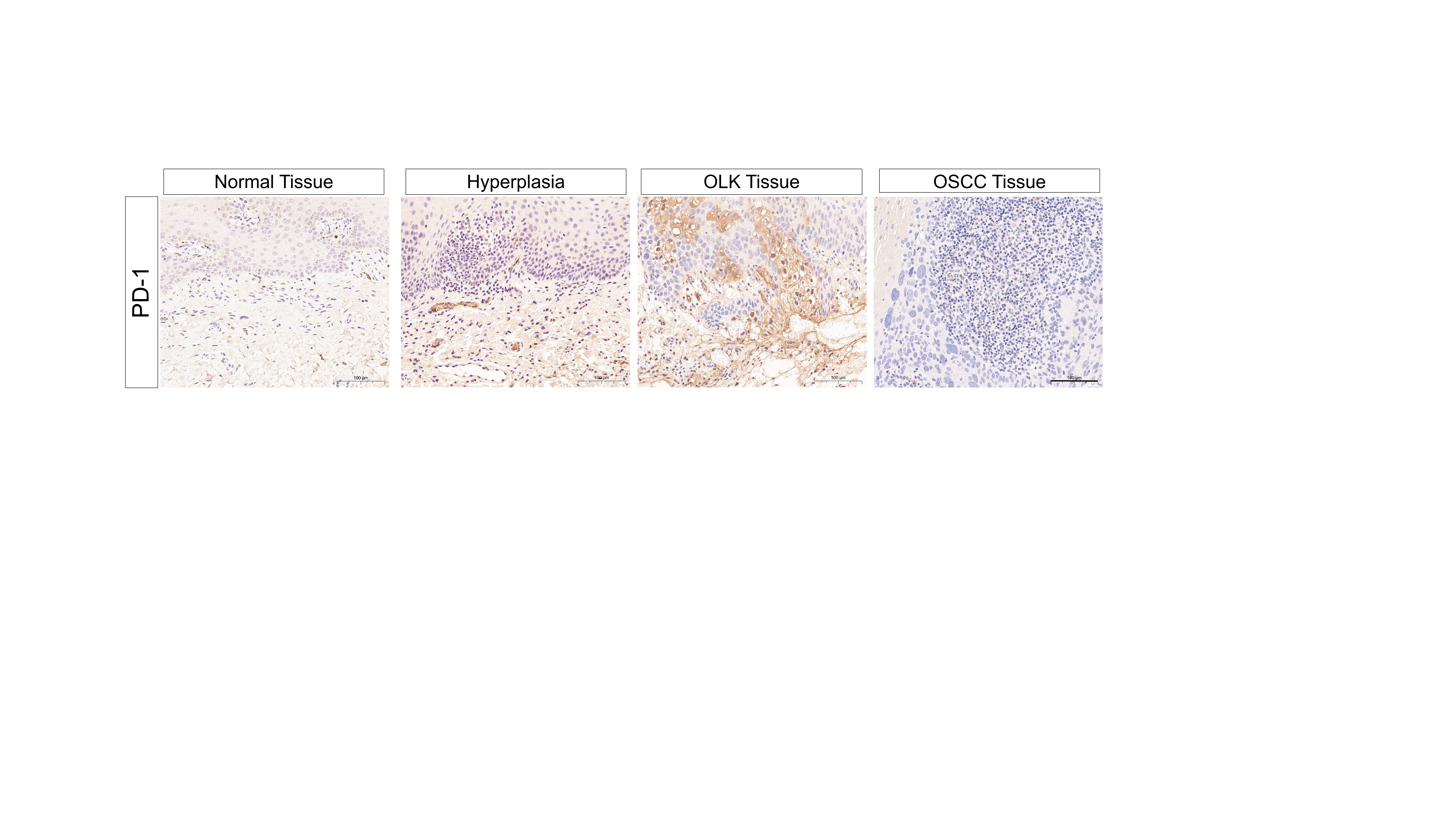
***

**Fig. S4 The expression of PD-1 in different tissues**

***Figure S5.***


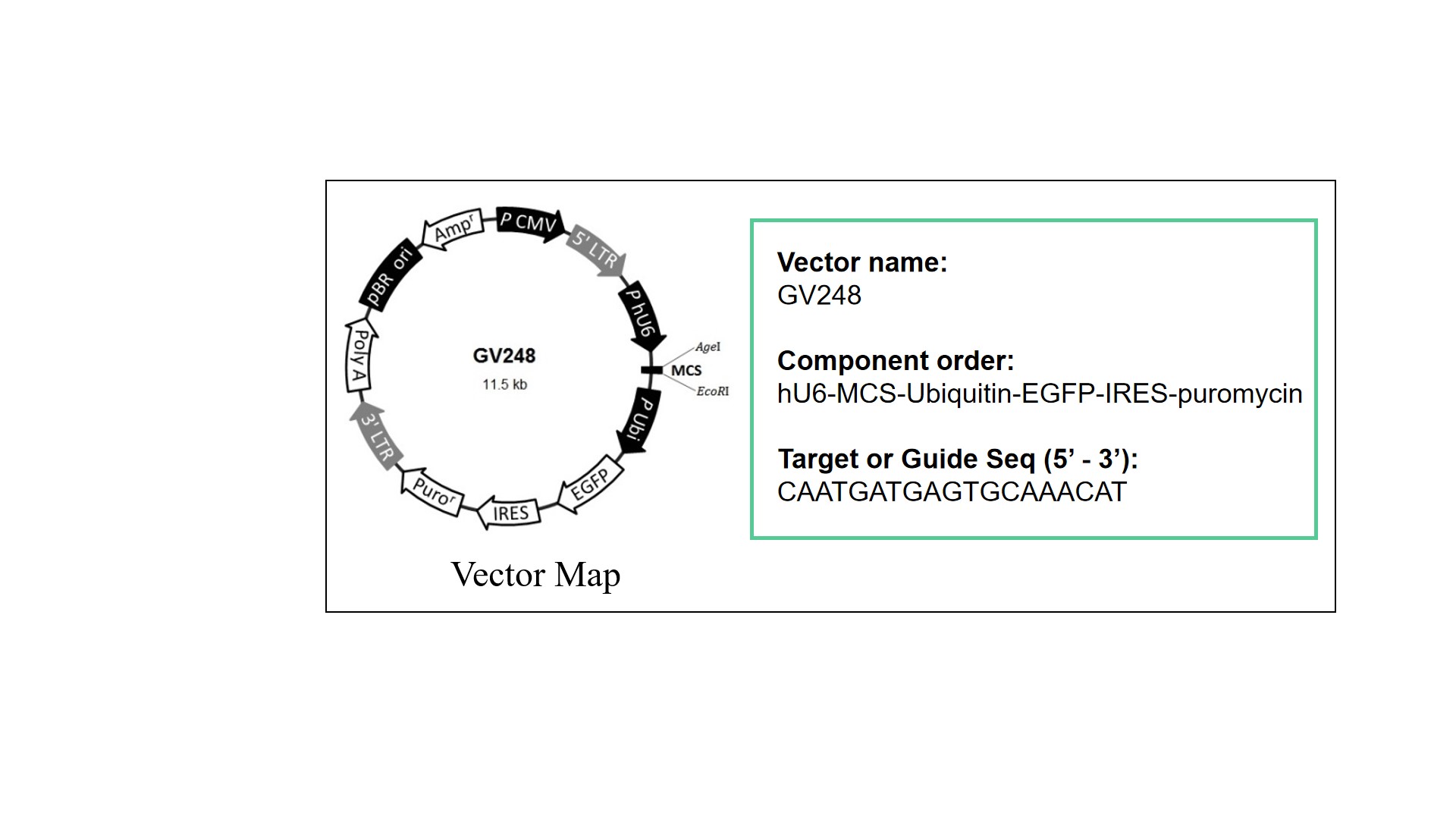


**Fig. S5. The information of the lentivirus carring FEN1 gene.**
